# A positive role for senescence in reproduction?

**DOI:** 10.18632/aging.100538

**Published:** 2013-02-28

**Authors:** Sumati Rajagopalan, Eric O. Long

**Affiliations:** Laboratory of Immunogenetics, NIAID, NIH, Rockville, MD 20852, USA

The induction of cellular senescence and the associated growth arrest are well accepted as a mechanism to block the development of cancer due to neoplastic transformation. Ironically, the senescence associated secretory profile (SASP) of senescent cells has been shown to promote cancer and other age related pathologies [1]. At the same time, the SASP is beneficial in tissue repair, such as during wound healing. Reconciling these contradictory outcomes from an evolutionary standpoint has been a challenge and the theory of antagonistic pleiotrophy has been evoked to address this [2]. According to this theory, genes that display antagonistic pleiotrophy (control some beneficial traits and some detrimental traits) favor successful reproduction early in life but have deleterious effects later in life. We have recently identified cellular senescence as a mechanism by which innate lymphocytes can promote vascular remodeling through their senescence secretome [3]. Such a new role for senescence would favor reproduction by promoting the neovascularization that occurs in early pregnancy to guarantee adequate blood supply to the developing fetus.

Natural killer (NK) cells are white blood cells that are important in immune defense and in reproduction. Their role in immunity is based on their ability to kill stressed or infected cells and to secrete various cytokines and chemokines, which promote resistance to infections and regulate adaptive immune responses. The role of NK cells in reproduction is less well understood, although several genetic association studies have correlated NK cell receptors and their HLA ligands with disorders of pregnancy such as recurrent spontaneous abortions or preeclampsia [4]. What is clear is that NK cells are abundant at the maternal-fetal interface; their role at this site does not appear to involve cytotoxic effector functions.

In early pregnancy, upon implantation of the blastocyst in the maternal uterine wall, fetal trophoblast cells from the outer layer of the embryo invade the maternal decidua (the pregnant uterus) and can be seen intermingled with NK cells at this site. In the first weeks of pregnancy, these trophoblast cells express HLA-G, a major histocompatibility complex (MHC) molecule that is not usually present on somatic cells [4]. Our earlier work showed that NK cells express CD158d (KIR2DL4), a receptor that binds HLA-G and signals intracellularly from endosomes for a proinflammatory and proangiogenic response [5]. Signaling for this response involved the phosphorylation of the serine-threonine kinase Akt by the DNA damage signaling kinase DNA-PKcs, and NF-κB activation [6]. The surprising involvement of a kinase belonging to DNA damage response (DDR) signaling pathways led us to test for the induction of the cyclin kinase inhibitor, p21, a key determinant of senescence [3]. NK cells stimulated with either an agonist antibody for CD158d or its ligand, soluble HLA-G, upregulated p21 and this was blocked in the presence of inhibitors of DNA-PKcs. These NK cells underwent characteristic changes in cell size and shape and staining for β-galactosidase was consistent with senescence-associated β-galactosidase (SABG). They were also arrested at the G0/G1 stage of the cell cycle and showed no evidence of either proliferation or apoptosis.

Microarray analysis of NK cells stimulated through CD158d showed evidence of both senescence and SASP [3]. Furthermore, the secretome of these cells was capable of inducing vascular permeability and angiogenesis, consistent with a potential role in vascular remodeling. These studies were carried out using primary, resting NK cells from human peripheral blood. Uterine NK cells isolated from first trimester abortions are not suitable for studies on their regulation and function in early pregnancy, as the early events of activation, including stimulation by trophoblast-derived HLA-G have already occurred. However, to test the hypothesis that such cells would show evidence of a senescence response, we did a retrospective analysis of microarray data from decidual NK cells isolated from first trimester abortions as compared to resting peripheral blood NK cells [7]. We found evidence of a senescence signature, which was validated by gene set enrichment analysis (GSEA) with published microarray data from cells undergoing oncogene-induced senescence [8].

What are the implications of this senescence response in early pregnancy? NK cells at the implantation site would be sensors of trophoblast invasion by responding to the HLA-G secreted by these invading fetal cells. Endosomal signaling by CD158d in response to HLA-G would induce a senescent state in NK cells. This would initiate a sustained secretory response that would promote the vascular remodeling required of the mother early in pregnancy. In this way, the resulting SASP can shape the local environment to increase vascularization required for the growth of the fetus. In this context, senescence would be a positive force in promoting reproductive success and all the disparate features of senescence ranging from the growth arrest to the secretome would be focused towards a common function of favoring a successful pregnancy.

Thus, although classically associated with aging and progression to the end of a lifespan, senescence may play a key role at the very beginning of this journey by fostering an environment that can sustain the growth and development of the early embryo.
